# Integrative modeling of multi-omics data to identify cancer drivers and infer patient-specific gene activity

**DOI:** 10.1186/s12918-016-0260-9

**Published:** 2016-02-11

**Authors:** Ana B. Pavel, Dmitriy Sonkin, Anupama Reddy

**Affiliations:** Graduate Program in Bioinformatics, Boston University, 24 Cummington Mall, Boston, 02215 MA USA; Section of Computational Biomedicine, Boston University School of Medicine, 72 East Concord Street, Boston, 02118 MA USA; Novartis Institutes for Biomedical Research, 250 Massachusetts Ave, Cambridge, 02139 MA USA; Duke University Medical Center, Durham, 27708 NC USA

**Keywords:** Fuzzy logic modeling, Gene activity, Oncogene, Tumor suppressor, Drug sensitivity, Colorectal cancer subtypes

## Abstract

**Background:**

High throughput technologies have been used to profile genes in multiple different dimensions, such as genetic variation, copy number, gene and protein expression, epigenetics, metabolomics. Computational analyses often treat these different data types as independent, leading to an explosion in the number of features making studies under-powered and more importantly do not provide a comprehensive view of the gene’s state. We sought to infer gene activity by integrating different dimensions using biological knowledge of oncogenes and tumor suppressors.

**Results:**

This paper proposes an integrative model of oncogene and tumor suppressor activity in cells which is used to identify cancer drivers and compute patient-specific gene activity scores. We have developed a Fuzzy Logic Modeling (FLM) framework to incorporate biological knowledge with multi-omics data such as somatic mutation, gene expression and copy number measurements. The advantage of using a fuzzy logic approach is to abstract meaningful biological rules from low-level numerical data. Biological knowledge is often qualitative, thus combining it with quantitative numerical measurements may leverage new biological insights about a gene’s state. We show that the oncogenic and altered tumor suppressing state of a gene can be better characterized by integrating different molecular measurements with biological knowledge than by each data type alone. We validate the gene activity score using data from the Cancer Cell Line Encyclopedia and drug sensitivity data for five compounds: BYL719 (PIK3CA inhibitor), PLX4720 (BRAF inhibitor), AZD6244 (MEK inhibitor), Erlotinib (EGFR inhibitor), and Nutlin-3 (MDM2 inhibitor). The integrative score improves prediction of drug sensitivity for the known drug targets of these compounds compared to each data type alone. The gene activity scores are also used to cluster colorectal cancer cell lines. Two subtypes of CRCs were found and potential cancer drivers and therapeutic targets for each of the subtypes were identified.

**Conclusions:**

We propose a fuzzy logic based approach to infer gene activity in cancer by integrating numerical data with descriptive biological knowledge. We compute general patient-specific gene-level scores useful to determine the oncogenic or tumor suppressor status of cancer gene drivers and to cluster or classify patients.

**Electronic supplementary material:**

The online version of this article (doi:10.1186/s12918-016-0260-9) contains supplementary material, which is available to authorized users.

## Background

Cancer is a complex genetic and genomic disease driven by many different molecular mechanisms. Cancer studies have employed high throughput technologies to profile genes in multiple different dimensions. Genetic variation, copy number, gene and protein expression, epigenetics, and metabolomics are the most commonly studied molecular types of data. Computational analyses commonly evaluate multiple data types as a set of independent features [1]. This leads to a multi-fold increase in the number of features. The statistical limitations in high-dimensional data, where the number of samples is considerably smaller than the number of measurements for each sample, is discussed in [2]. Moreover, each data type represents an incomplete snapshot of a biological process and does not provide a comprehensive view of a gene state. In this paper, we propose an integrative approach of multiple data types to model the activity of oncogenes and tumor suppressors and characterize cancer gene drivers.

An oncogene is a gene that has the potential to cause cancer and it is often mutated in tumor cells. A tumor suppressor is a gene that protects a cell from cancer. Oncogenes present gain of function alterations (GoF), while inactivated tumor suppressor genes present loss of function (LoF) alterations. A cancer driver gene contains driver gene mutations, but may also contain passenger gene mutations with no effect in cancer. Driver mutations are identified based on their pattern of mutations across samples [3]. Well studied oncogenes are recurrently mutated at the same amino acid positions, while tumor suppressors are mutated through protein-truncating alterations throughout their length [3].

We will refer, throughout the paper, to a gene’s potential to cause cancer as a gain of function (GoF)/loss of function (LoF) *gene activity*.

Large studies like the The Cancer Genome Atlas (TCGA) [4], International network of cancer genome projects (ICGC) [5], Cancer Cell Line Encyclopedia (CCLE) [6] have profiled thousands of tumors for many different data types (exome sequencing, copy number, expression, methylations, etc.). Data portals, such as cBioPortal [7], which contain data from these large cancer studies, and which are widely used by the community, display an integrative view of all data types for a given gene. These plots are very powerful as they show a comprehensive view of different mechanisms by which genes can be aberrant in cancer. Our aim in this paper is to develop a computational framework to provide an integrative activity score of a gene.

Other studies have shown that incorporating biological knowledge into model building improves prediction of breast cancer survival [8] and glioblastoma subtypes [9]. Using probabilistic inference, the method proposed in [9] predicts the degree to which the activity of a pathway is altered in a patient. A gene is modeled as a set of interconnected variables which encode for expression, copy number and protein levels.

Other studies have shown that integration of multiple molecular data types may better characterize the disease. TCGA studies [10] and [11] evaluate multiple data types independently to characterize lung cancer. An integrative approach for predicting the tumor suppressor functional status of a gene is presented in [12]. Using CCLE dataset, the authors show that *bi-allelic* inactivation of tumor suppressors may occur through genetic mechanisms (loss of function mutation, copy number loss, or loss of heterozygosity) or epigenetic mechanisms (promoter methylation or histone modification) or a combination of the two.

Integrative approaches through network based analysis have been previously developed to predict driver genes. An example is OncoIMPACT framework which nominates patient-specific driver genes based on their phenotypic impact [13]. This approach uses gene interaction networks to associate mutations with changes in cell state, such as transcriptome, proteome, epigenome or metabolome. Another example is the analysis pipeline proposed in [14] which integrates genomic and transcriptomic alterations from whole-exome and RNA sequence data and functional data from protein function prediction and gene interaction networks. This method predicts functional implications of mutated potential driver genes found within and across patients with breast cancer.

In this paper we present a novel approach based on Fuzzy Logic Modeling (FLM) to infer patient-specific GoF/LoF *gene activity* by integrating multiple molecular data types in a single gene-level score. We use matched gene expression, copy number and mutation data from CCLE and integrate them using biological knowledge about oncogenes and tumor suppressors. Other existing approaches identify cancer drivers by assessing only one data type such as mutation frequency [3], or by correlating mutations with other data types or phenotypes. However, the two-dimensional correlation models the relationship of two variables across patients, while the proposed methodology allows integrating any number of data types at the patient level. Moreover, the FLM score is general and independent of a particular group of patients or phenotype in the dataset. Other methods [9, 15] use probabilistic inference to integrate different types of molecular data with pathway-level information in a patient-specific activity score. These methods depend on prior information about the curated pathway and the gene interactions, assuming a local pathway context for a given gene. The method in [15] models the interaction of a mutated gene with the abundance levels of the upstream and downstream genes, while our method captures the global change of a gene based on its own mutation status and abundance level. Differently from the existing approaches, we use descriptive and intuitive knowledge about cancer drivers to combine multiple data types at the gene-level in a unified patient-specific score. The FLM scores are computed for every gene, therefore these could further be integrated at a pathway-level using graphical models, similarly to [9, 15]. The proposed scores can be used to (*i*) determine the oncogenic or tumor suppressor status of a gene by assessing the sign of the score; (*ii*) determine the activity level of an oncogene or tumor suppressor by assessing the magnitude of the score; (*iii*) classify samples to predict a specific phenotype; (*iv*) cluster samples to identify subtype specific gene drivers; (*v*) reduce the feature space by 3-fold to increase the statistical power of sample stratification. Our approach is general, and can be extended by adding other data types and descriptive rules about known biological processes. We validate the proposed methodology by computing gene activity scores to predict drug sensitivity. In addition we classify genes into oncogenes and tumor suppressors and validate the status of known cancer genes. We also use the activity scores to cluster colorectal cancer (CRC) cell lines.

We show that *gene activity* can be better described by integrating different molecular measurements than by analyzing each data type alone. To the authors’ knowledge, this is the first study in CCLE data showing that the GoF activity of a gene is characterized by a combination of mutation status, expression level and copy number changes. Moreover, by integrating three measurements in a single score, we are able to reduce the feature space by 3-fold, and therefore increase the statistical power of sample stratification. In addition, the proposed *gene activity* score can be used to highlight potential cancer gene drivers to improve therapeutic strategies.

## Method

### Fuzzy logic modeling

Fuzzy logic is an artificial intelligence paradigm inspired by how people intuitively measure variables such as temperature, noise, taste. For example, people generally tend to think of temperature in terms of cold/warm/hot categories instead of in degrees. The categories are loosely defined (fuzzy) and subjective, but still meaningful. Among few applications of fuzzy logic are image stabilization, the control of robots and automations, medical decision support systems, etc. Previous studies have shown that fuzzy logic can be used to build robust gene classifiers [16], to eliminate redundant information from microarray data [17] and to detect gene regulatory networks from microarray data [18]. General concepts and applications about fuzzy logic in Bioinformatics can also be found in [19].

**Fig. 1:**
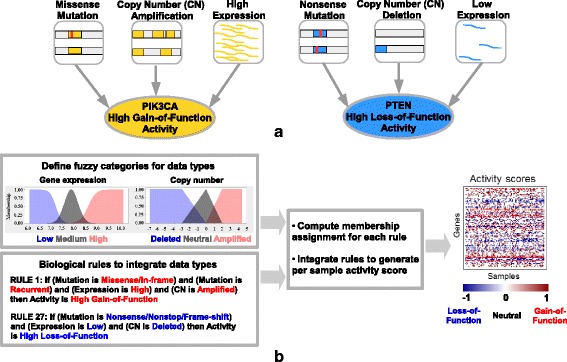
Inferring *gene activity* by integrating different data types and biological knowledge. **a** Example showing how mutation, copy number and expression data are important for inferring the activity of PIK3CA (oncogene), and PTEN (tumor suppressor). **b** Schematic for Fuzzy Logic Modeling (FLM)

In this paper we present a novel approach using fuzzy logic to integrate multiple types of molecular data with expert curated biological rules. We integrate mutation, gene expression and copy number data to generate sample-specific *gene activity* scores (Fig. 1a). We chose fuzzy logic as a modeling framework due to its flexibility of incorporating biological knowledge described by high-level rules with data measurements.

A fuzzy logic system is a rule-based model in which categories are defined within a numeric range. These categories do not have to be disjoint. Each numeric value is assigned a degree of belief to each category. Categories are defined as functions modeling the degree of uncertainty. These functions are called *membership functions* and estimate the degree to which numeric values belong to each category. The fuzzy system computes the output by evaluating a set of rules defined on the categories. Once the rules are evaluated, the result is then *defuzzified* (converted back to a numeric value) by averaging across the output of the rules. In this paper we will use the *centroid* method for defuzzification. Figure 1b illustrates the categories, membership functions and workflow of the proposed fuzzy logic model.

For example, gene expression data can be divided into categories such as *low*, *medium* and *high*, which enables us to abstract away from the numeric data and define inference rules based on the categories (Fig. 1b). The framework is flexible and allows adding any number of data types or rules. The inference rules can easily be extended or modified without changing the meaning of the categories and their numeric range.

The output of the proposed system is a per sample continuous score [–1, 1]. Positive values indicate that the gene presents a gain of function (GoF) with a level of activity between 0 (lowest) and 1 (highest), while negative values indicate that the gene presents a loss of function (LoF) with a level of inactivation between 0 (lowest) and –1 (highest) in a given sample.

We tested our approach on publicly available data that was downloaded from the Cancer Cell Line Encyclopedia (CCLE)Cancer Cell Line Encyclopedia (CCLE) [6]. We included in our analysis 675 cell lines and a number of 1547 protein-coding genes with matched somatic mutation, copy number and gene expression measurements. We used somatic mutation data which was generated via hybrid capture exome sequencing by [6]. About 1600 protein-coding genes were sequenced based on their known or potential involvement in tumor biology, as described in [6]. We also used copy number (genome-wide human Affymetrix SNP Array 6.0) and gene expression (Affymetrix Human Genome U133 Plus 2.0 arrays) normalized data from [6]. More details about generating and normalizing the CCLE data can be found in [6].

JFuzzyJFuzzy, an open source Java-based software, was used to implement and test the proposed fuzzy logic system.

### Defining rules based on biological knowledge of oncogenes and tumor suppressors

Rules for oncogenic or gain of function (GoF) activity and tumor suppressor-like or loss of function (LoF) activity are defined. We consider the GoF and LoF types of mutations presented in [3]: a recurrent missense or in-frame indel may indicate a GoF, while a nonsense, nonstop, frame-shift indel or splice site mutation may indicate a LoF. We hypothesize that a gene with a recurrent GoF mutation which also has a higher expression and/or copy number will be more active in cancer compared to lower expression or deletions. For example mutations, as well as amplifications and over-expression of EGFR gene are known to be implicated in many epithelial cancers, such as lung cancer, glioblastoma, colorectal cancer, etc. [20, 21]. Similarly, a gene with a LoF mutation and low expression and/or copy number will be a more active cancer driver than a highly expressed LoF mutant (the non-mutated allele may be expressed in enough amount to perform the gene’s function). An example is TP53 gene which can loose its tumor supressor function either by LoF mutations or by deletions or under-expression [12, 22, 23].

For those genes that have no or silent mutations, we consider that loss in expression or copy number may indicate LoF, while a gain in expression or copy number levels may indicate a GoF activity. However expression and copy number do not contribute symmetrically to LoF and GoF scores because low expression or deletions are more likely to produce a deactivated tumor suppressor, compared to the symmetric situation with high expression or amplification for activated oncogenes. We take this into account in our rules.

The following shows the two extreme rules in our FLM system. We have 28 more rules that define the spectrum between these two extremes (Table 1).

- GoF rule (rule 1): If a variant is activating (missense, in-frame indel) and is recurrent (same position) and the expression is high and the copy number is amplified then the gene is a very activating GoF.

**Table 1:** Fuzzy rules

RULE 1: IF ((Mutation IS Missense_Mutation) or (Mutation IS In_Frame_Del) or (Mutation IS In_Frame_Ins)) and (Recurrence IS recurrent) and (Expression IS high) and (CN IS amplified) THEN Gene_activity IS high_GoF;
RULE 2: IF ((Mutation IS Missense_Mutation) or (Mutation IS In_Frame_Del) or (Mutation IS In_Frame_Ins)) and (Recurrence IS recurrent) and (Expression IS high) and (CN IS neutral) THEN Gene_activity IS high_GoF;
RULE 3: IF ((Mutation IS Missense_Mutation) or (Mutation IS In_Frame_Del) or (Mutation IS In_Frame_Ins)) and (Recurrence IS recurrent) and (Expression IS high) and (CN IS deleted) THEN Gene_activity IS GoF;
RULE 4: IF ((Mutation IS Missense_Mutation) or (Mutation IS In_Frame_Del) or (Mutation IS In_Frame_Ins)) and (Recurrence IS recurrent) and (Expression IS medium) and (CN IS amplified) THEN Gene_activity IS high_GoF;
RULE 5: IF ((Mutation IS Missense_Mutation) or (Mutation IS In_Frame_Del) or (Mutation IS In_Frame_Ins)) and (Recurrence IS recurrent) and (Expression IS medium) and (CN IS neutral) THEN Gene_activity IS GoF;
RULE 6: IF ((Mutation IS Missense_Mutation) or (Mutation IS In_Frame_Del) or (Mutation IS In_Frame_Ins)) and (Recurrence IS recurrent) and (Expression IS medium) and (CN IS deleted) THEN Gene_activity IS low_GoF;
RULE 7: IF ((Mutation IS Missense_Mutation) or (Mutation IS In_Frame_Del) or (Mutation IS In_Frame_Ins)) and (Recurrence IS recurrent) and (Expression IS low) and (CN IS amplified) THEN Gene_activity IS high_GoF;
RULE 8: IF ((Mutation IS Missense_Mutation) or (Mutation IS In_Frame_Del) or (Mutation IS In_Frame_Ins)) and (Recurrence IS recurrent) and (Expression IS low) and (CN IS neutral) THEN Gene_activity IS GoF;
RULE 9: IF ((Mutation IS Missense_Mutation) or (Mutation IS In_Frame_Del) or (Mutation IS In_Frame_Ins)) and (Recurrence IS recurrent) and (Expression IS low) and (CN IS deleted) THEN Gene_activity IS no_effect;
RULE 10: IF ((Mutation IS Missense_Mutation) or (Mutation IS In_Frame_Del) or (Mutation IS In_Frame_Ins)) and (Recurrence IS non_recurrent) and (Expression IS high) and (CN IS amplified) THEN Gene_activity IS GoF;
RULE 11: IF ((Mutation IS Missense_Mutation) or (Mutation IS In_Frame_Del) or (Mutation IS In_Frame_Ins)) and (Recurrence IS non_recurrent) and (Expression IS high) and (CN IS neutral) THEN Gene_activity IS low_GoF;
RULE 12: IF ((Mutation IS Missense_Mutation) or (Mutation IS In_Frame_Del) or (Mutation IS In_Frame_Ins)) and (Recurrence IS non_recurrent) and (Expression IS high) and (CN IS deleted) THEN Gene_activity IS low_GoF;
RULE 13: IF ((Mutation IS Missense_Mutation) or (Mutation IS In_Frame_Del) or (Mutation IS In_Frame_Ins)) and (Recurrence IS non_recurrent) and (Expression IS medium) and (CN IS amplified) THEN Gene_activity IS low_GoF;
RULE 14: IF ((Mutation IS Missense_Mutation) or (Mutation IS In_Frame_Del) or (Mutation IS In_Frame_Ins)) and (Recurrence IS non_recurrent) and (Expression IS medium) and (CN IS neutral) THEN Gene_activity IS low_GoF;
RULE 15: IF ((Mutation IS Missense_Mutation) or (Mutation IS In_Frame_Del) or (Mutation IS In_Frame_Ins)) and (Recurrence IS non_recurrent) and (Expression IS medium) and (CN IS deleted) THEN Gene_activity IS no_effect;
RULE 16: IF ((Mutation IS Missense_Mutation) or (Mutation IS In_Frame_Del) or (Mutation IS In_Frame_Ins)) and (Recurrence IS non_recurrent) and (Expression IS low) and (CN IS amplified) THEN Gene_activity IS low_GoF;
RULE 17: IF ((Mutation IS Missense_Mutation) or (Mutation IS In_Frame_Del) or (Mutation IS In_Frame_Ins)) and (Recurrence IS non_recurrent) and (Expression IS low) and (CN IS neutral) THEN Gene_activity IS no_effect;
RULE 18: IF ((Mutation IS Missense_Mutation) or (Mutation IS In_Frame_Del) or (Mutation IS In_Frame_Ins)) and (Recurrence IS non_recurrent) and (Expression IS low) and (CN IS deleted) THEN Gene_activity IS no_effect;
RULE 19: IF ((Mutation IS Frame_Shift_Ins) or (Mutation IS Frame_Shift_Del) or (Mutation IS Nonsense_Mutation) or (Mutation IS Nonstop_Mutation) or (Mutation IS Splice_Site)) and (Expression IS high) and (CN IS amplified) THEN Gene_activity IS low_LoF;
RULE 20: IF ((Mutation IS Frame_Shift_Ins) or (Mutation IS Frame_Shift_Del) or (Mutation IS Nonsense_Mutation) or (Mutation IS Nonstop_Mutation) or (Mutation IS Splice_Site)) and (Expression IS high) and (CN IS neutral) THEN Gene_activity IS LoF;
RULE 21: IF ((Mutation IS Frame_Shift_Ins) or (Mutation IS Frame_Shift_Del) or (Mutation IS Nonsense_Mutation) or (Mutation IS Nonstop_Mutation) or (Mutation IS Splice_Site)) and (Expression IS high) and (CN IS deleted) THEN Gene_activity IS high_LoF;
RULE 22: IF ((Mutation IS Frame_Shift_Ins) or (Mutation IS Frame_Shift_Del) or (Mutation IS Nonsense_Mutation) or (Mutation IS Nonstop_Mutation) or (Mutation IS Splice_Site)) and (Expression IS medium) and (CN IS amplified) THEN Gene_activity IS LoF;
RULE 23: IF ((Mutation IS Frame_Shift_Ins) or (Mutation IS Frame_Shift_Del) or (Mutation IS Nonsense_Mutation) or (Mutation IS Nonstop_Mutation) or (Mutation IS Splice_Site)) and (Expression IS medium) and (CN IS neutral) THEN Gene_activity IS LoF;
RULE 24: IF ((Mutation IS Frame_Shift_Ins) or (Mutation IS Frame_Shift_Del) or (Mutation IS Nonsense_Mutation) or (Mutation IS Nonstop_Mutation) or (Mutation IS Splice_Site)) and (Expression IS medium) and (CN IS deleted) THEN Gene_activity IS high_LoF;
RULE 25: IF ((Mutation IS Frame_Shift_Ins) or (Mutation IS Frame_Shift_Del) or (Mutation IS Nonsense_Mutation) or (Mutation IS Nonstop_Mutation) or (Mutation IS Splice_Site)) and (Expression IS low) and (CN IS amplified) THEN Gene_activity IS high_LoF;
RULE 26: IF ((Mutation IS Frame_Shift_Ins) or (Mutation IS Frame_Shift_Del) or (Mutation IS Nonsense_Mutation) or (Mutation IS Nonstop_Mutation) or (Mutation IS Splice_Site)) and (Expression IS low) and (CN IS neutral) THEN Gene_activity IS high_LoF;
RULE 27: IF ((Mutation IS Frame_Shift_Ins) or (Mutation IS Frame_Shift_Del) or (Mutation IS Nonsense_Mutation) or (Mutation IS Nonstop_Mutation) or (Mutation IS Splice_Site)) and (Expression IS low) and (CN IS deleted) THEN Gene_activity IS high_LoF;
RULE 28: IF ((Mutation IS No_Mutation) and ((Expression IS low) or (CN IS deleted))) THEN Gene_activity IS LoF;
RULE 29: IF ((Mutation IS No_Mutation) and ((Expression IS high) or (CN IS amplified))) THEN Gene_activity IS low_GoF;
RULE 30: IF ((Mutation IS No_Mutation) and (Expression IS medium) and (CN IS neutral)) THEN Gene_activity IS no_effect;
- LoF rule (rule 27): If a variant is inactivating (nonsense, nonstop, frame-shift indels, splice site) and the expression is low and copy number is deleted then the gene is a very inactivating LoF.

In the case of TP53 variants, missense mutations (generally GoF mutations) drive the gene’s LoF [3, 12, 23–25]. We have added special rules for TP53 to take this into account.

The proposed inference rules are shown in Table 1 and are also available in Additional file 1.

### Defining fuzzy categories for data types

For gene expression, we defined three fuzzy categories by dividing the expression values into three quantiles and computing mean and standard deviation (*std*) of each group. The *medium* level is defined by a Gaussian membership function. The *low* and *high* levels are sigmoid functions with the inflection points equal to the quantile’s mean. The slopes of the sigmoid functions were approximated with the slope of a Gaussian shape: ${slope_{\textit {sigmoid}} = {\sqrt {8 \cdot ln(2)} \over std_{\textit {gaussian}}}}$. For copy number, we defined identical shapes of membership functions for every gene (triangle for *neutral* level and trapezoid for *amplified* and *deleted* levels). Eight types of mutations are considered (missense, in-frame insertion, in-frame deletion, nonsense, nonstop, frame-shift insertion, frame-shift deletion, splice site). A variant is recurrent if it is present in more than 1 % of the samples at the same position. Additional file 1 is an example of a FCL (Fuzzy Control Language)FCL (Fuzzy Control Language) file which contains the definitions of membership functions and fuzzy rules.

### Computing activity score for each variant

The activity score is computed for each variant by applying the set of defined rules. The activity score is modeled by seven membership functions (Table 1 and Additional file 1), corresponding to the following activity categories: *high GoF* (Sigmoid membership function), *GoF*, *low GoF*, *no effect*, *low LoF*, *LoF* (Gaussian membership functions) and *high LoF* (Sigmoid membership function). The membership assignments of the rules are then aggregated by union. The integrated membership assignment is then converted back into a numeric value (deffuzified) using the centroid method. A score in [–1,1] interval is generated; 1 means maximum GoF, while –1, maximum LoF.

### Computing gene-level activity scores and labeling of genes as GoF/LoF

A gene usually presents multiple variants. FLM scores are computed for each variant. To summarize the scores at the gene-level we compute two different scores for each gene and each sample: GoF score (maximum across all GoF variants), LoF score (minimum across all LoF variants). Finally, the gene is labeled as GoF/LoF using the following algorithm: if the majority of the mutated samples (>50 *%*) have *GoF*>|*LoF*|, then the gene is labeled as GoF and the GoF percentage is selected as the gene-level score; similarly, if the majority of the mutated samples (>50 *%*) have |*LoF*|>*GoF*, then the gene is labeled as LoF and the LoF percentage is selected as the gene-level score. To restrict the classification to genes that are more likely to be drivers in CCLE, we considered to classify those genes that are mutated in more than 1 % of the samples (a total number of 1288 genes).

## Results and discussion

### Computing the FLM gene activity scores on the Cancer Cell Line Encyclopedia

We used 675 cell lines with matched gene expression (Affymetrix U133 Plus 2.0), copy number (Affymetrix SNP6.0 arrays) and somatic mutation (hybrid capture exome sequencing) data from the Cancer Cell Line Encyclopedia (CCLE) [6].

We propose a set of inference rules based on biological knowledge that integrate information about mutation type, mutation frequency, level of expression and copy number amplifications or deletions (Table 1 and Additional file 1).

**Fig. 2:**
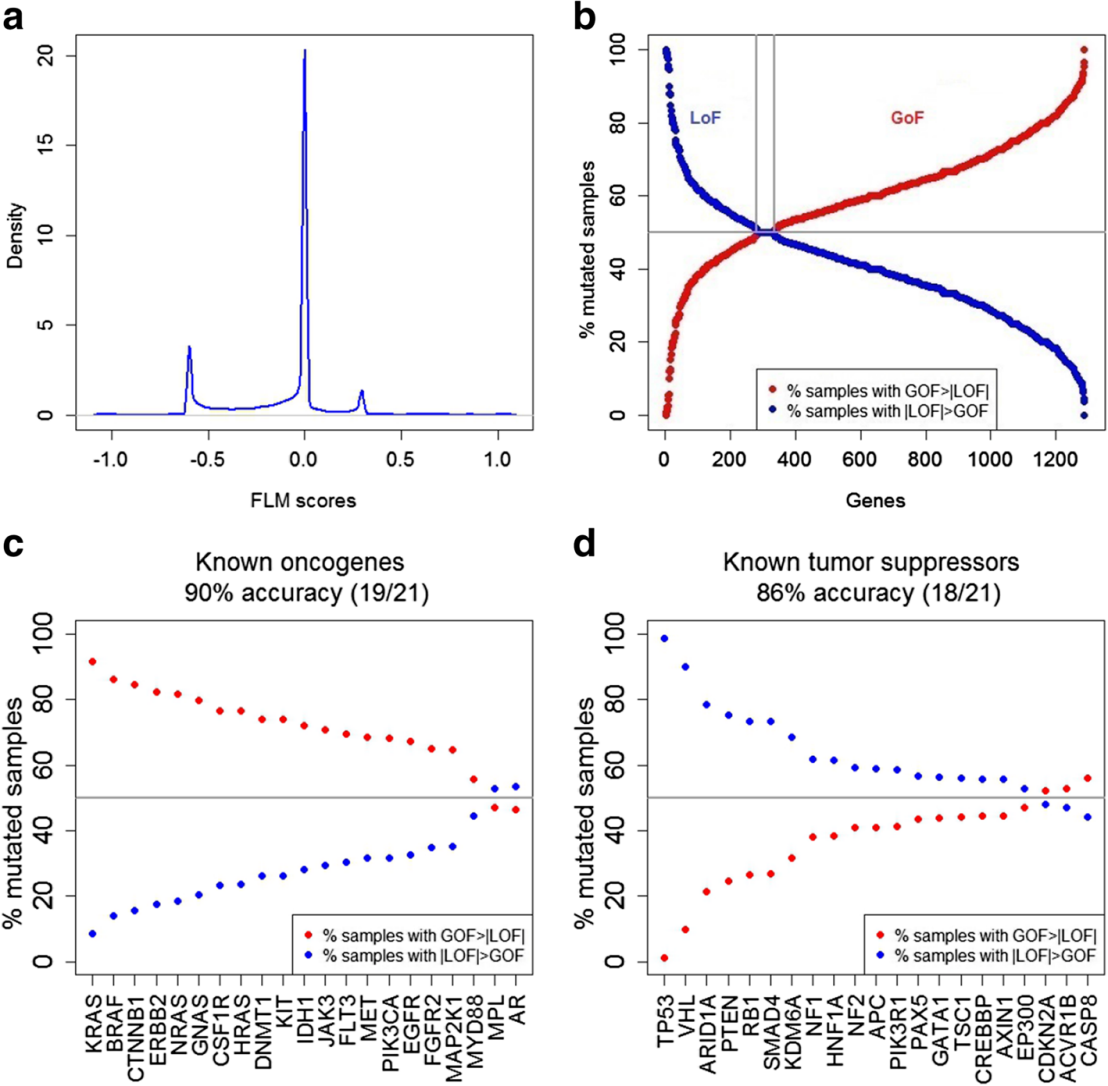
*Gene activity* scores and inferred GoF/LoF status using Fuzzy Logic Modeling. **a** Distribution of GoF and LoF activity scores across all genes and all samples. **b** For each gene that presents mutations in CCLE (more than 1 % of the samples), two scores are computed (GoF and LoF gene score). GoF gene score is computed as the percentage of mutated samples with *G* *o* *F*>|*L* *O* *F*|. LoF gene score is computed as the percentage of mutated samples with |*L* *o* *F*|>*G* *o* *F*. A gene is classified as GoF (oncogene) if the GoF gene score is >50 *%* or as LoF (tumor suppressor) if the LoF gene score is >50 *%*. **c** Known oncogenes [3] were correctly predicted by our method with an accuracy of 90 % (19/21). **d** Known oncogenes [3] were correctly predicted by our method with an accuracy of 86 % (18/21). Note that the known oncogenes and tumor suppressors were restricted to those that were found to be mutated in the CCLE at >1 *%* frequency

The distribution of the GoF and LoF scores across all analyzed genes and samples is presented in (Fig. 2a). This distribution is trimodal with peaks at high LoF, no activity and high GoF scores.

A number of 1288 genes are assigned GoF and LoF gene scores based on the GoF and LoF percentages in mutated samples, as described in “Computing gene-level activity scores and labeling of genes as GoF/LoF”. The gene score >50 *%* selects the final gene status (Fig. 2b). The GoF and LoF gene scores are available in Additional file 2.

### FLM identifies known oncogenes and tumor suppressors

Next, we compared the proposed GoF/LoF classification with oncogene/tumor suppressor status proposed in [3]. The known oncogenes and tumor suppressors were restricted to those that were found to be mutated in CCLE at >1 *%* frequency. Figures 2c and d show that our approach correctly classified well known oncogenes and tumor suppressors that were also correctly classified by the method proposed in [3]. The accuracy for GoF clasification was 90 % (19/21), and 86 % (18/21) for LoF, respectively. We conclude that the FLM-based approach of classifying GoF/LoF is consistent with [3]. The method proposed by [3] computes a gene-level score across variants. In addition, FLM approach provides a per sample *gene activity* score that can further be used for sample stratification.

### Gene activity scores improve drug sensitivity predictions

The proposed fuzzy rules and the computed *gene activity* scores were validated by predicting drug sensitivity of cell lines in the CCLE. CCLE provides drug perturbation data which allows us to assess gene activity of specific targeted compounds. Although several compounds were tested in CCLE [6], the mechanisms of action and gene targets are unknown for most of them. To validate our approach, we considered those compounds which directly target a driver gene, known to mutate in cancer, or a gene that directly interacts with a mutant driver gene. We selected one target-compound from each family by excluding those with multiple targets and those which do not interact with a specific mutant gene, since their effect may not directly reflect the activity of the driver gene used as a predictor of sensitivity. Therefore, we used in our analysis targeted compounds profiled in [6], such as PLX4720 (BRAF inhibitor), AZD6244 (MEK inhibitor), Erlotinib (EGFR inhibitor), and Nutlin-3 (MDM2 inhibitor). In addition, we analyzed the drug sensitivity data for BYL719 (PIK3CA inhibitor) from [26]. We tested the prediction performance of drug sensitivity for these compounds using their known driver gene targets.

Drug sensitivity of cell lines can be measured by different metrics, such as the area under the dose response curve (*ActivityArea* or *ActArea*), high-concentration effect level (*A*_*max*_), the transitional concentration (*EC*_50_), the concentration at which the drug response reached an absolute inhibition of 50 % (*IC*_50_) or a combination of these metrics [6, 26]. Although these metrics are theoretically equivalent and correlated with the response of the cells to the drug, it has been shown that some of them work better for certain compounds [6]. Moreover, the thresholds which separate the three classes of cell lines, sensitives, intermediates and resistants, are estimated from the response curve and usually differ from one compound to another. Sensitivity calls have already been validated for BYL719 and Nutlin-3 by other published studies. BYL719 sensitive/resistant classes have been defined using a combination of thresholds for *A*_*max*_ and *EC*_50_ metrics in [26], while Nutlin-3 sensitive/resistant classes have been defined using thresholds for *IC*_50_ metric in [12]. For the other compounds we estimated the sensitivity calls by applying a Gaussian Mixture Model on *ActArea* variable, as recommended in [6]. The thresholds that define the two populations of sensitive/resistant cell lines, were computed based on the means (*μ*_1_,*μ*_2_), standard deviations (*σ*_1_,*σ*_2_) and the intersection point of the two Gaussian distributions (*X*). Resistant cell lines were defined as those with *ActArea*< min(*X*,*μ*_1_+*σ*_1_), while the sensitives, as those with *ActArea*> max(*X*,*μ*_2_+*σ*_2_).

Table 2 shows the list of compounds, the genes they target, their sensitivity predictor genes, and the thresholds used to define their sensitivity calls. The genes that are able to predict drug sensitivity could be direct targets of a drug or its off-target effects. For example, Nutlin-3 compound inhibits MDM2 gene which is an inhibitor of TP53 [22, 27, 28]. A cell line may be sensitive to Nutlin-3 if TP53 is not mutated (it normally functions as a TSG) and it is expressed in enough amount (TP53 expression is not suppressed by interactions other than MDM2). Figure 4c shows higher LoF level in the sensitive cell lines.

**Table 2:** Gene targets and the predictors of sensitivity for the compounds

Compounds	Direct gene targets	Known sensitivity predictors	Sensitivity threshold	Resistance threshold
PLX4720	BRAF	BRAF mutation	*A* *c* *t* *A* *r* *e* *a*≥2.05	*A* *c* *t* *A* *r* *e* *a*≤0.47
AZD6244	MEK	BRAF mutation	*A* *c* *t* *A* *r* *e* *a*≥3.02	*A* *c* *t* *A* *r* *e* *a*≤0.69
Erlotinib	EGFR	EGFR mutation	*A* *c* *t* *A* *r* *e* *a*≥1.74	*A* *c* *t* *A* *r* *e* *a*≤0.42
Nutlin-3	MDM2	TP53 mutation	*I* *C*50≤4.26	*I* *C*50≥6.94
		TP53 expression		
BYL719	PIK3CA	PIK3CA mutation	*E* *C*50≤3.04	*E* *C*50>3.04
			*A* *m* *a* *x*≤–30	*A* *m* *a* *x*>–30

#### Evaluating the AUC on FLM activity scores for each sensitivity predictor gene

**Fig. 3:**
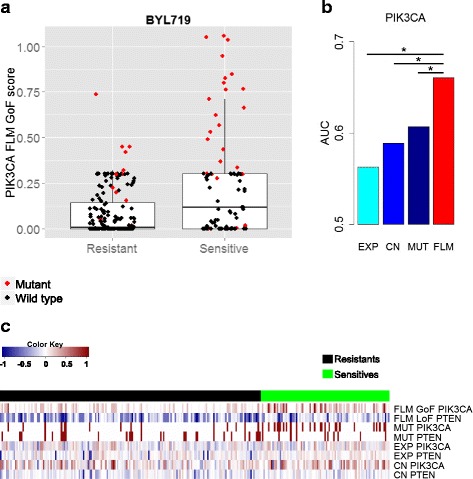
FLM *gene activity* scores improve prediction of BYL719 drug sensitivity compared to using expression, mutation and copy number data separately. **a** Boxplot for PIK3CA FLM scores vs. BYL719 (PIK3CA inhibitor) sensitivity. BYL719 sensitive group has higher activity scores compared to the resistant group (*t-test p* <10^−4^). Even within the PIK3CA missense mutants (colored in red), we see that FLM GoF scores are higher in sensitive compared to resistant group (*t-test p* <0.0008). **b** Using PIK3CA FLM GoF scores to predict sensitivity, the AUC significantly improved compared to expression, mutation and copy number data separately, *p*<0.05. We denote by * the significance level of 0.05. **c** Heatmap showing the FLM activity scores for PIK3CA, PTEN and the individual data types. All values are scaled between [–1, 1]. Note that our algorithm correctly labeled PIK3CA as a GoF gene, and PTEN as a LoF gene, consistent with their classification in the literature. The color bar on top indicates the sensitivity groups for the samples (*green = sensitive*, *black = resistant*). The combined predictor of PIK3CA GoF scores and PTEN LoF scores significantly improves performance compared to combinations of individual data types, *p*<0.009

**Fig. 4:**
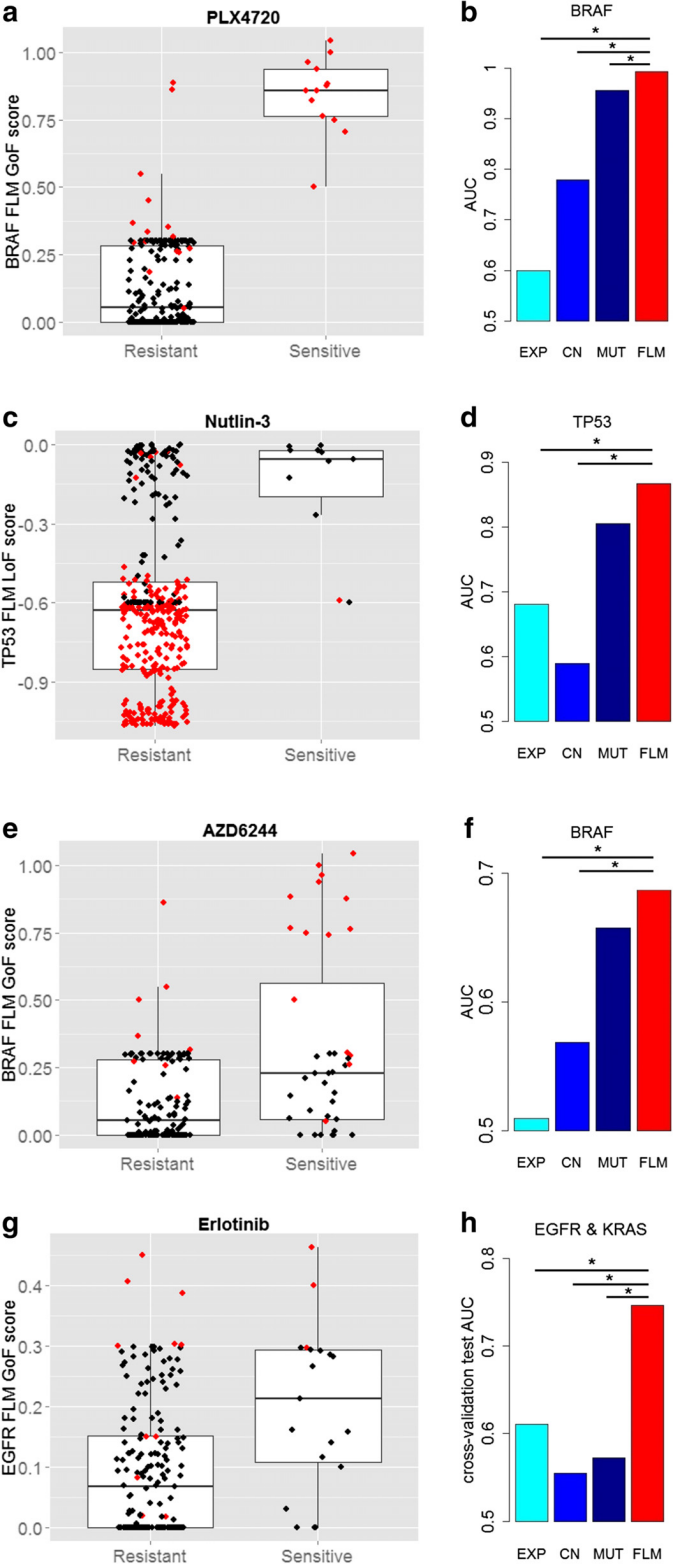
FLM *gene activity* scores differentiate the sensitive vs. resistant groups better than the relevant mutations (colored red) in each compound: **a** PLX4720, **c** Nutlin-3, **e** AZD6244, **g** Erlotinib. FLM scores improve prediction of drug sensitivity compared to gene expression, somatic mutation and copy number data separately: **b** PLX4720, *p*<0.00002, **d** Nutlin-3, *p*<0.06, **f** AZD6244, *p*<0.22, **h** Erlotinib using EGFR-KRAS predictor, *p*<0.01. We denote by * the significance level of 0.05

We computed the Area Under the Receiver Operating Characteristic Curve (AUC) to evaluate the prediction performance of drug sensitivity for each gene predictor and each data type. For each gene, we computed the AUC metric on each data type separately, as well as on the sample-specific FLM scores. We chose AUC as a valid comparison metric of the prediction performance for the different data types because it can be measured on both binary (such as mutation) and continuous (such as gene expression, copy number and FLM activity scores) values. Then, *bootstrap* test [29] available in pROC R package [30] was applied in order to compare the AUCs. For all the five compounds, the performance of FLM activity scores was improved compared to each of the data types (expression, copy number and mutation) as shown in Figs. 3 and 4. We tested the sensitivity predictor gene of each compound (Table 2). The FLM scores significantly improved prediction compared to all feature types for BYL719 (PIK3CA gene): *p*<0.05, PLX4720 (BRAF gene): *p*<0.00002 and Nutlin-3 (TP53 gene): *p*<0.06. For AZD6244 (BRAF gene) the improvement of FLM score was significant with respect to expression and copy number data (*p*<0.01), while the increase in performance was not significant compared to mutation data (*p*=0.22). For Erlotinib (EGFR gene) the improvement of FLM score was significant with respect to mutation and copy number data (*p*<0.04), while the increase in performance was not significant compared to expression data (*p*=0.13). In Figs. 3 and 4, the significance level of 0.05 was denoted by *.

#### Comparing the FLM scores with numeric integration of mutation, gene expression and copy number data

Next, we tested whether the FLM scores outperform the numeric integration of the three data types by comparing the performance of the FLM scores with the performance of the three data types used as a set of classification features. Logistic regression is a commonly used technique for classification of samples based on a set of features [31–33]. This algorithm aggregates the numerical values of the three data types, regardless of the biological knowledge captured by the fuzzy rules, and learns from the training data to distinguish sensitives from resistants. The logistic regression model (glmnet) was ran within k-fold cross-validation (*k*=5). Similarly to the cross-validation procedure described in [34], each sample was assigned to a *test fold* exactly once. Then, we used the test prediction scores of all samples to compute the overall *test* AUC. We compared the AUC of the glmnet classifier with a single FLM feature to the AUC of the glmnet classifier with three features of different data types by using *bootstrap* test [29, 30]. The integrative FLM feature performed significantly better compared to the numeric integration of the three features, for AZD6244 (BRAF gene): *p*=0.02, PLX4720 (BRAF gene): *p*=0.02 and Erlotinib (EGFR gene): *p*=0.04. For BYL719 (PIK3CA gene) and Nutlin-3 (TP53 gene) respectively, the difference in AUCs was not statistically significant.

#### Building multiple genes classifiers on FLM activity scores

A combined predictor for BYL719 involving PIK3CA and PTEN genes was proposed in [26]. Cell lines with PTEN LoF are less likely to respond to the drug. Therefore, to further evaluate the power of FLM activity scores, we test the prediction performance of the two genes on FLM activity scores, mutation, gene expression and copy number data. A logistic regression model (glmnet) was ran within k-fold cross-validation (*k*=5) to test the AUC of the 2-genes predictors. Similarly to the cross-validation procedure described in [34], each sample was assigned to a *test fold* exactly once. Then, we used the test prediction scores of all samples to compute the overall *test* AUC. The AUC of each data type was then compared to the AUC of the FLM scores by using *bootstrap* test [29, 30]. The PIK3CA-PTEN predictor increased in performance and FLM scores were significantly better than using each of the individual data types, *p*<0.009.

Next, we performed a similar analysis for Erlotinib, using EGFR and KRAS combined predictors. The prediction was significantly improved by FLM activity scores compared to the other data types, *p*<0.01 (Fig. 4h). KRAS is situated downstream of EGFR [35] and cell-lines with KRAS mutation are less likely to respond to Erlotinib. The EGFR-KRAS predictor increased in performance and FLM scores were significantly better than using each of the individual data types.

Therefore, we have shown that FLM is a robust gene-level score which can be used to build better predictors of drug sensitivity compared to those developed on each data type alone. In addition, FLM scores are biologically meaningful and more importantly, the knowledge based information enriches the numeric data measurements.

### Using the FLM activity scores to cluster colorectal cancers

In this section we present the utility of FLM activity scores for clustering cancers. FLM integrates multiple data types (somatic mutation, gene expression and copy number) to compute gain/loss of function scores for each gene. The feature space is reduced by 3-fold, therefore clustering cell lines using the FLM scores may enable a more accurate stratification of cancers compared to using each data type separately. Additionally, this also enables us to directly identify cancer drivers of each subtype.

Colorectal cancer is a heterogeneous and genetically complex disease with tumors bearing a high mutation load. To date, no gene expression signature was proven to be reliable for stratification in clinical practice. A recent study of 1100 colorectal cancer patients reported three main molecularly distinct subtypes (CCS1, CCS2 and CCS3) using gene expression data [36]. CCS1 was associated with MSI (micro-satellite instable), CIMP (CpG island methylator phenotype), CCS2 was associated with CIN (chromosomal instability) and KRAS/TP53 mutations, while CCS3 was a novel finding with poor prognosis and was associated with serrated adenomas.

**Fig. 5:**
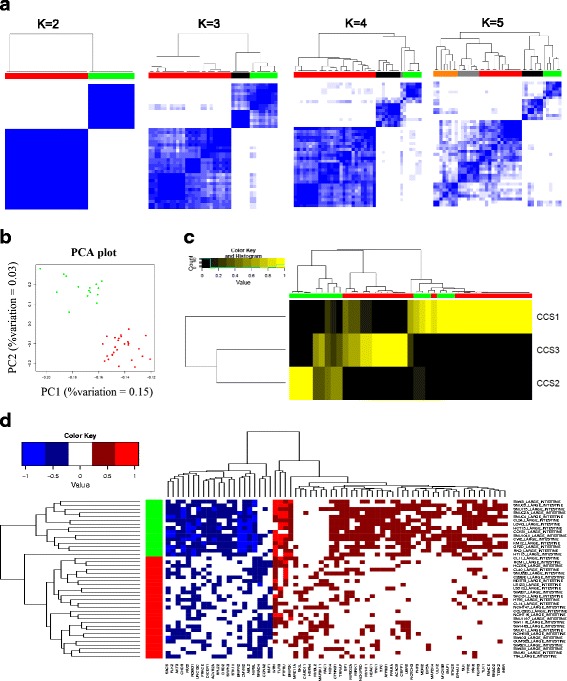
Identifying unsupervized clusters in colorectal cancer and finding differential *gene activity* within each cluster. **a** Consensus matrix for *K*=2,3,4,5, using k-means clustering on colorectal cell lines. The consensus matrices show that there are two distinct subtypes which are stable even when K is increased. **b** Principal component analysis (PCA) plot of the FLM *gene activity* scores for 42 colorectal cancer cell lines. Colors indicate the two subtypes found using consensus clustering. **c** Subtypes found by FLM in CCLE are validated by comparing with subtypes in TCGA [36]. CCS2 is correlated with cluster 2 (green), while cluster 1 is split between CCS1 and CCS3. **d** Heatmap of the significantly differential *gene activity* scores (*Student’s t-test*, *F* *D* *R*<0.05) which differentiate the two FLM subtypes

We used FLM scores of the 1547 genes to stratify the colorectal cancer cell lines into clusters based on their activity scores. We ran consensus clustering [37, 38] on 42 colorectal cancer cell lines from the CCLE and identified 2 clusters (Fig. 5a, b). We compared the FLM clusters with the previously obtained CCS clusters [36]. The heatmap (Fig. 5c) shows the posterior probability of association with each of the CCS clusters. We can notice a clear overlap between the FLM cluster 2 (green) vs. CCS2. Most likely we do not see a difference between clusters 1 and 3 because of reduced sample size. Another reason may be that FLM identifies oncogenes and tumor suppressors which are active across multiple subtypes. Also, by using activity scores instead of gene expression data we may detect a lineage effect that drives CCS3.

Then, by using the gene activity scores, we identified statistically significantly differential active genes (*Student’s t-test*, *FDR*<0.05) which differentiated the two clusters (Fig. 5d). We can directly identify cancer drivers from these results. Cluster 1 (red) has suppressed apoptosis (ACVR2A [39], ABL2 [40], PLK2 [41]), while Cluster 2 (green) has activated oncogenes like MDM2 [22] and FGFR3 [42]. We show that using FLM scores we can validate subtypes found in larger studies as well as identify cancer drivers in each of the subtypes.

## Conclusions

We propose and validate a novel approach for integrating molecular data to infer patient-specific activity of oncogenes and tumor suppressor genes using fuzzy logic modeling. We show that gain of function or loss of function of a gene can be better characterized by integrating different molecular measurements with biological knowledge than by analyzing each data type separately.

The main advantage of the fuzzy logic framework is that the data level is separated from the inference level, therefore expert knowledge can be incorporated into the system, regardless of the data representation. Another important advantage is the generality of the FLM scores which is computed for each patient and each gene. Therefore, the FLM score can be used to classify genes into oncogenes and tumor suppressors, determine the level of activity for each oncogene and tumor suppressor, and moreover classify or cluster the patients.

FLM is a flexible method to incorporate measurements obtained from different platforms using knowledge based rules. Noise from each individual data type is reduced by categorizing the data, and by integrating multiple data types together. Samples can be stratified using an integrated activity score which better captures the state of each gene. Moreover, reducing the feature space by a factor of three enhances the statistical power of the sample stratification studies, which is especially helpful in data sets of limited sample size.

The novel FLM activity scores improves the prediction of drug response for five different compounds, compared to mutation status, gene expression and copy number data. In addition, we are also able to significantly improve prediction of BYL719 drug sensitivity using a combined predictor of PIK3CA GoF score and PTEN LoF score and for Erlotinib using a combined predictor of EGFR and KRAS GoF scores. Moreover, the performance of FLM scores for clasifying drug responders outperforms the numeric integration of the three different data types for AZD6244, PLX4720 and Erlotinib. Therefore, we conclude that the proposed integrative approach is biologically meaningful and more importantly, the knowledge based information enriches the numeric data measurements.

Another interesting observation revealed by our method is that oncogenic activity of a gene is characterized by both mutation status and its gene expression levels.

We also validate the status of known oncogenes and tumor suppressor genes. Our method highlights additional potential oncogenes and tumor suppressors, which will further be explored. The inference rules can be easily adapted to capture particular gene behaviors that are exceptions to the proposed general set of rules, as we have shown for TP53 case. We plan to further extend the rules and incorporate more expert knowledge, especially for the case of exceptions.

Moreover, we present an application of the FLM scores for sample stratification and subtype discovery. We use the FLM gene scores as clustering features of the colorectal cell lines. We are able to identify colorectal subtypes which were previously obtained in a larger sample size study. We also highlight potential gene drivers associated with each subtype.

The proposed activity scores can be utilized for the prediction of cells’ response to different perturbations such as drugs or shRNAs and may help in identifying cancer driver genes. The patient-specific gene-level scores can also be used for subtype discovery and stratification of the samples, as we have shown with the clustering of colon cancers. The FLM scores can generally be utilized to classify or cluster samples, enhancing the statistical power of such studies by reducing the feature space. Activated oncogenes and inactivated tumor suppressors can be identified within each group.

The proposed framework is flexible and can further be extended to incorporate other data measurements which may provide additional information about the gene’s state, such as the methylation status, the expression of microRNA regulators or transcription factors. In future work we also plan to further explore the FLM scores by studying other CCLE compounds with more complex mechanisms of action, such as multiple downstream or unknown gene targets. In addition, exploring different sensitivity levels and correlating them with FLM scores will be considered. Furthermore, we plan to extend this approach to predict the activity of molecular pathways. For example, the proposed FLM activity scores can be used as the nodes of a gene network. Similar approaches of pathway level integration were proposed in [9] and [15]. We aim to integrate the *gene activity* scores with pathway-level information to model gene interactions and evaluate the impact of the FLM scores on the activity of molecular pathways.

## Availability of supporting data

The results published in this paper are based upon the publicly available data from the Cancer Cell Line Encyclopedia [6] (doi:10.1038/nature1100310.1038/nature11003): http://www.broadinstitute.org/ccle/home.

## Additional files

Additional file 1
**An example of the defined membership functions and general fuzzy rules in FCL (Fuzzy Control Language) format.** (FCL 8.15 kb)

Additional file 2
**GoF and LoF gene scores for those genes that are mutated in more than 1 % of the samples.** (CSV 37.9 kb)

## Abbreviations

AUC: area under the receiver operating characteristic curve
CCLE: cancer cell Line encyclopedia
CIN: chromosomal instability
CRC: colorectal cancer
FDR: false discovery rate
FLM: fuzzy logic modeling
GoF: gain of function
ICGC: international network of cancer genome projects
LoF: loss of function
ONG: oncogene
TCGA: the cancer genome atlas
TSG: tumor suppressor gene
